# Airport Site Selection of Tehran, Iran

**Published:** 2019-06

**Authors:** Maryam KIANISADR

**Affiliations:** Department of the Environment, College of Basic Sciences, Hamedan Branch, Islamic Azad University, Hamedan, Iran

## Dear Editor-in-Chief

Locating airport requires a wider range of studies than other transportation models because aviation has been one of the vibrant industries and predicting required future information of that is very complicated (1). Therefore, the current study checked and developed the criteria for locating airports in Tehran Province, Iran. Besides the desirability of various zones of the province in terms of airport establishment were evaluated.

Integration of Multiple Attribute Decision Making (MCDM) methods and GIS were used. MCDM is one of mathematical models and refers to an approach of solving problem used in order to choose an option of a limited number of items (2).

Using the analytic network process (ANP) has been significantly limited in processing the information due to human finite capacity. The Technique for Order of Preference by Similarity to Ideal Solution (TOPSIS) method can provide the requirement of network comparisons and capacity limitation will not dominate the process (3). Tehran Province with its center Tehran and an area of 12981 km^2^, is located between 34 and 36.5 degrees’ north latitude and 50 to 53 degrees’ east longitude. The population of this province had been 12425000 people in 2014. The center of this province is Tehran City.

**Fig. 1:**
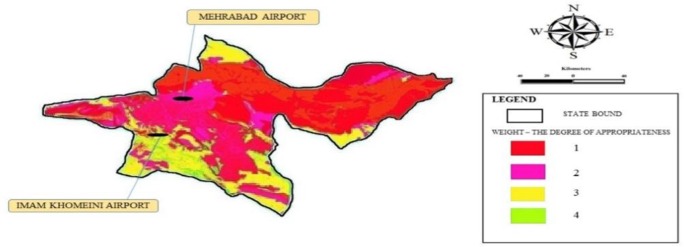
Zoning the location of airport in Tehran Province

Tehran Province has two airports of Imam Khomeini and Mehrabad. Effective environmental criteria on airport site selection were scaled in 5 classes. Then, TOPSIS and Super Decision software were used for determining the weight of criteria. To integrate the layers and multi-criteria decision making, TOPSIS methods and weighted overlay method were used. Figure 1 shows the obtained results of weighted overlay and TOPSIS methods in locating airport of Tehran Province.

The mentioned map has some zones with the degree of mismatch 1), poor 2), medium 3) and right 4). The results show the necessity of wide zoning for airports establishment in an area or province. The function of combining methods of MCDM and Arc GIS in recent locations has been confirmed in many studies (4, 5).
